# Efficacy and safety of remimazolam compared with propofol in hypertensive patients undergoing breast cancer surgery: a single-center, randomized, controlled study

**DOI:** 10.1186/s12871-023-02364-x

**Published:** 2023-12-12

**Authors:** Yaqi Huang, Ting Yan, Guiting Lu, Huirong Luo, Zhongmeng Lai, Liangcheng Zhang

**Affiliations:** 1 Department of Anesthesiology, Fujian Medical University Union Hospital, No. 29 Xin-Quan Road, Fuzhou, 350001 China; 2 Department of Anesthesiology, Fujian Medical University, Fuzhou, China

**Keywords:** Remimazolam, Propofol, Anesthesia, Hypertensive patient, Breast cancer Surgery, Hemodynamics

## Abstract

**Background:**

Remimazolam, as a novel anesthetic, has recently been shown to improve hemodynamic stability during anesthesia induction and maintenance; however, it has not been reported in the hypertensive population. This study aimed to compare the effects of remimazolam and propofol on hemodynamic stability in hypertensive patients undergoing breast cancer surgery.

**Methods:**

We enrolled 120 hypertensive patients undergoing breast cancer surgery in this prospective study and randomly allocated them to remimazolam (n = 60) or propofol (n = 60) groups. Anesthesia regimens were consistent between groups, except for the administration of remimazolam and propofol. Our primary outcome was the incidence of post-induction hypotension, which was either an absolute mean arterial pressure (MAP) < 60 mmHg or a > 30% relative drop in MAP compared to baseline within 20 min of induction or from induction to the start of surgery. Secondary outcomes included minimum MAP and MAP at different time points during anesthesia, the application of vasoactive drugs, adverse events, and the patient’s self-reported Quality of Recovery-40 scale for the day after surgery.

**Results:**

The incidence of post-induction hypotension was lower and the minimum MAP during induction was higher in the remimazolam group than those in the propofol group. There were no significant differences between the two groups in the remaining outcomes.

**Conclusion:**

Remimazolam is safe and effective in hypertensive patients undergoing breast cancer surgery. Induction with remimazolam in hypertensive patients may result in more stable hemodynamics than propofol.

**Trial registration:**

This study was registered at the Chinese Clinical Trials Registry (http://www.chictr.org.cn) on 03/12/2020, with registration number ChiCTR2000040579.

**Supplementary Information:**

The online version contains supplementary material available at 10.1186/s12871-023-02364-x.

## Background

Hypertension is a common cardiovascular disease in China, with a prevalence of 44.7%, and its prevalence is gradually increasing every year [1, 2]. Hypertensive patients have poor cardiovascular system compensatory capacity and are prone to dramatic hemodynamic fluctuations during anesthetics and surgical procedures. Severe perioperative hypertension or hypotension can cause or worsen myocardial ischemia [3], and lead to complications such as stroke and renal failure [4], which negatively impact patient prognosis [5]. Therefore, rational selection of anesthetics is crucial for hypertensive patients.

Propofol is the most commonly used intravenous anesthetic agent in clinical practice due to its rapid onset and recovery, short continuous infusion half-life, and complete awakening. However, because of its inhibitory effect, particularly on the respiratory and circulatory systems, propofol has been designated as a drug with a narrow therapeutic index [6–8], and its clinical application, especially for hypertensive patients, has been limited.

Remimazolam is a novel, ultra-short-acting benzodiazepine with sedative and hypnotic effects [9]. It is organ-independently metabolized to inactive metabolites, has rapid induction and recovery, and is antagonized by flumazenil [10–12]. Although remimazolam’s anesthetic sedation effect is comparable to that of propofol, the incidence of intraoperative hypotension and respiratory depression is lower than that of propofol [13]. In theory, remimazolam could be ideal for hypertensive patients undergoing general anesthesia.

However, relevant randomized controlled studies to validate the efficacy and safety of remimazolam in hypertensive patients are still lacking. Therefore, this study aimed to evaluate the hemodynamic stability, occurrence of adverse events, and quality of postoperative recovery after the administration of remimazolam in general anesthesia for hypertensive patients undergoing breast cancer surgery. Propofol was administered to the control group.

## Methods

### Design and patients

We conducted a single-center, double-blinded, randomized controlled clinical trial among hypertensive patients aged 40–86 years who were scheduled for breast cancer surgery under general anesthesia at Fujian Medical University Union Hospital between September 2021 and June 2022. The inclusion criteria included patients with (1) a body mass index of 18–30 kg/m^2^, and (2) American Society of Anesthesiologists physical status I–III. Exclusion criteria included (1) allergy to drugs used in this study; (2) baseline blood pressure > 180/110 mmHg; (3) breast-conservation patients; (4) use of benzodiazepines or opioids within 1 month; (5) craniocerebral injury and intracranial hypertension; (6) history of mental illness; and (7) contraindications to remimazolam use (such as myasthenia gravis, schizophrenia, and severe depression).

Patients who provided consent were randomly assigned in a 1:1 ratio to the remimazolam or propofol group. Randomization was computer-generated using Epical 2000 software. Due to the significantly different properties of the two anesthetics, anesthesiologists could not be blinded to the group assignment. However, the allocation was completely concealed from follow-up investigators and participants.

### Anesthesia methods

Hypertensive patients were defined as individuals who had a prior diagnosis of hypertension before admission or who met the diagnostic criteria for hypertension after admission, regardless of their medication use or pattern. A preoperative visit was routinely performed on the day before the operation to explain the study to the patient and obtain written informed consent from the patient. Baseline blood pressure was measured by averaging three consecutive blood pressure measurements. After entering the operating room, the electrocardiogram, pulse oxygen saturation, noninvasive blood pressure, and the bispectral index (BIS) were monitored, and peripheral venous access was routinely obtained. Radial artery catheters were inserted under lidocaine infiltration anesthesia, and arterial blood pressure was continuously monitored and recorded using the arterial sensor once a minute.

In the remimazolam group, general anesthesia was induced with intravenous injections of remimazolam (Jiangsu Hengrui Medicine Co. Ltd., approval number: 210721AK) at 0.3 mg/kg, sufentanil at 0.4 µg/kg, and cisatracurium at 0.2 mg/kg. Anesthesia was maintained with remimazolam (0.3 mg/kg/h), remifentanil (5 µg/kg/h), and sevoflurane (0.5–1 MAC). In the propofol group, general anesthesia was induced with intravenous injections of propofol (Sichuan Guorui Pharmaceutical Co. Ltd., approval number: H20091713) at 2 mg/kg, sufentanil at 0.4 µg/kg, and cisatracurium at 0.2 mg/kg. Anesthesia was maintained with propofol (2 mg/kg/h), remifentanil (5 µg/kg/h), and sevoflurane (0.5–1 MAC). The injection speed of remimazolam and propofol was controlled within 40 s during the induction of anesthesia [14]. After induction of anesthesia, patients were intubated and mechanically ventilated with a tidal volume of 6–8 mL/kg. The end-tidal carbon dioxide concentration was maintained at 35–45 mmHg by adjusting the ventilation frequency. BIS was maintained between 40 and 60, and mean arterial pressure (MAP) was maintained within 30% of baseline blood pressure by adjusting sevoflurane concentrations and the corresponding administration of vasoactive drugs (urapidil and ephedrine). Atropine was administered if the heart rate was < 45 beats/min. The sevoflurane was discontinued 20 min before the end of the surgery, and the fresh gas flow was adjusted to 5 L/min. At the end of the surgery, the remifentanil, propofol, or remimazolam infusions were stopped, and sufentanil (5 µg) was administered. Neuromuscular blocks were reversed with atropine (0.5 mg) and neostigmine (1 mg) before tracheal extubation. The patients were transferred to the post-anesthesia care unit (PACU) for recovery.

### Outcome measures

We defined hypotension as a minimum MAP below 60 mmHg or a decrease of more than 30% from baseline. These hypotension definitions have previously been associated with adverse postoperative outcomes, even transient intraoperative episodes [4, 15, 16].

The main outcome of this study was the prevalence of “post-induction hypotension” (PIH, i.e., arterial hypotension occurring during the first 20 min after anesthesia induction or from anesthesia induction until the beginning of surgery.)

The secondary outcomes were as follows: (1) The incidence of hypotension and minimum MAP during the maintenance of anesthesia and in the PACU. (2) MAP at baseline (T_0_), 10 min after the induction of anesthesia (T_1_), and 10, 30, and 60 min after the surgery began (T_2_, T_3_, T_4_, respectively). (3) The administration of cardiovascular drugs to each patient. (4) Adverse events, including nausea and vomiting, blood pressure fluctuations, postoperative pain, postoperative delirium, and intraoperative awareness, were recorded in the PACU. Blood pressure fluctuations were considered to occur if MAP exceeded 30% of baseline. Postoperative pain was assessed using visual analog pain scales [17]. Intraoperative awareness was evaluated with a modified Brice interview [18, 19]. Postoperative delirium was estimated using the Nursing Delirium Screening Scale [20]. (5) The prognosis of patients one day after surgery was assessed using the scores of the Quality of Recovery-40 scale (QoR-40) [21].

Patient demographic and clinical parameters were retrieved, including age, body mass index, fasting time, preoperative fluid volume, intraoperative fluid volume, American Society of Anesthesiologists physical status, education level, and anesthesia time. Anesthesia time was defined as the time from anesthesia administration to anesthesia withdrawal.

### Sample size

PASS software (version 15, NCSS) was used to calculate the sample size. According to our preliminary findings, the incidence of hypotension after anesthesia induction was 0.633 and 0.367 in the propofol and remimazolam groups, respectively. We defined α as 0.05 and β as 0.2 and supposed that the rate of loss to follow-up was 10%. A sample size of 58 patients was required for each group. We finally included 120 patients for analysis in this study.

### Statistical methods

All data were expressed as mean ± SD, median (Q1; Q3), or number (percentage) as appropriate. The Shapiro–Wilk test was used to determine the normality of quantitative variables. Quantitative variables were compared between groups using the student *t*-test for normal distribution data and the Mann–Whitney U test for non-normal distribution data. The Chi-square test, or Fisher’s exact test, was used to compare qualitative variables between groups. An analysis of variance with two factors was used to analyze the MAP data. If a significant interaction was found, an appropriate post hoc analysis was used to determine the source of the significance. Multiple imputations were used to impute missing data, as MAP (in T3 and T4) were missing some data. All tests were two-sided, and *P* < 0.05 was considered statistically significant.

## Results

**Fig. 1:**
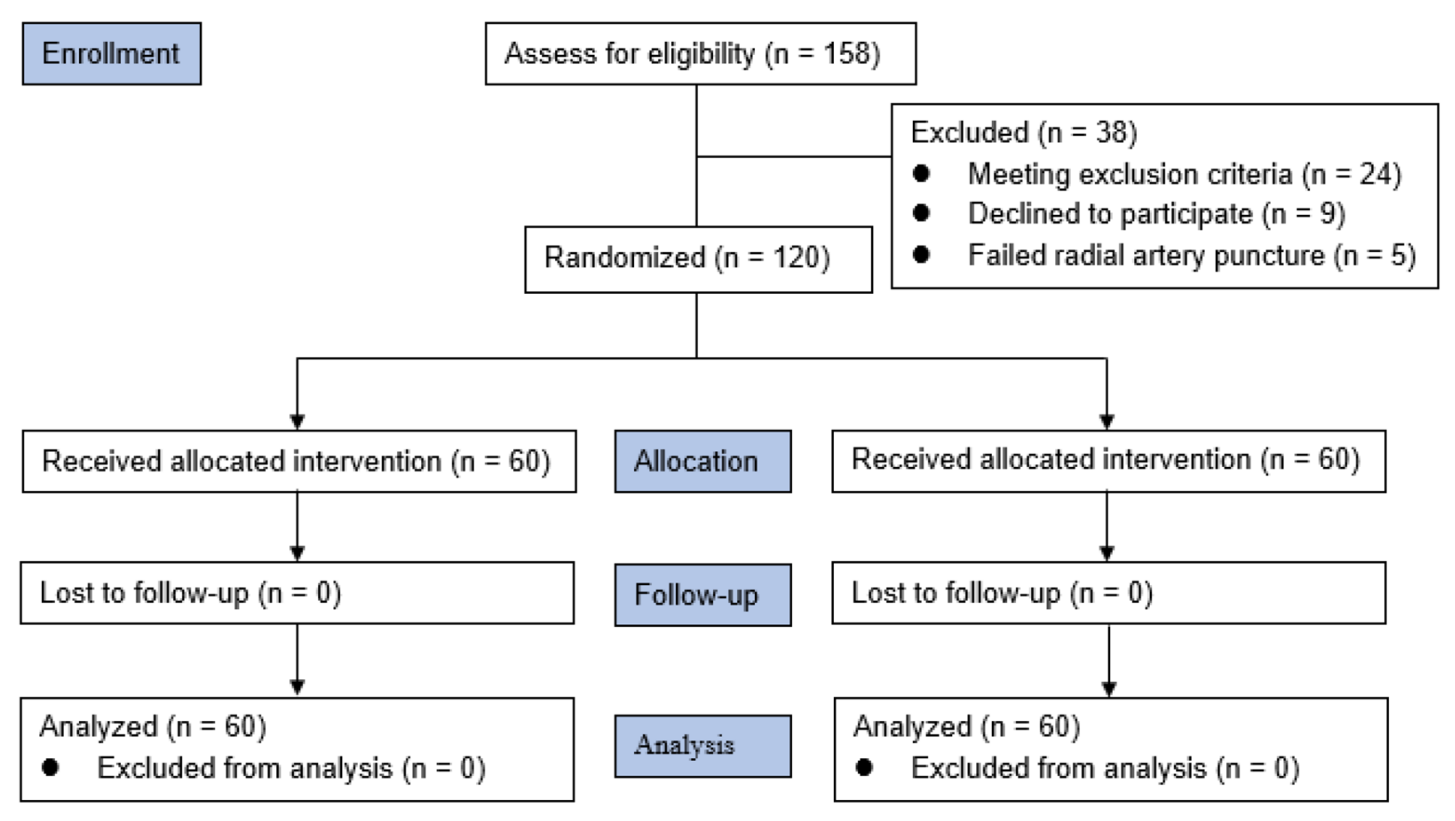
Consolidated Standards of Reporting Trials 2010 flow diagram

We initially screened 158 patients for eligibility between September 2021 and June 2022, of whom 38 were excluded, and 120 were randomly assigned to either the remimazolam or propofol groups. The details are presented in the flowchart (Fig. 1). The two groups’ demographic and clinical characteristics are compared in Table 1.

**Table 1:** Patient demographic and clinical parameters (n = 60 in each group)

	Propofol group	Remimazolam group	Chi-square value, t-value, or z-value	*P-*value
Age, (years)	63.8 ± 11.0	62.6 ± 8.9	0.696	0.490
BMI, (kg.m^− 2^)	24.8 ± 2.7	24.3 ± 2.6	1.004	0.320
Baseline SBP (mmHg)	141.9 ± 12.3	138.3 ± 15.8	1.396	0.165
Baseline DBP (mmHg)	77.8 ± 10.2	78.0 ± 12.1	-0.130	0.896
Baseline MAP (mmHg)	98.7 ± 9.2	98.3 ± 8.4	0.235	0.815
Fasting time (h)	14.6 ± 3.9	14.2 ± 4.2	0.522	0.600
Preoperative rehydration volume (mL)	500 (500, 800)	500 (400, 787)	-1.444	0.150
Intraoperative fluid volume (mL)	600 (550, 700)	700 (600, 800)	-1.599	0.120
ASA physical status, n (%)			2.143	0.200
II	24 (40.0)	32 (53.3)		
III	36 (60.0)	28 (46.7)		
Education level, n (%)			3.789	0.140
Elementary school and below	26 (43.3)	22 (36.7)		
Middle school	29 (48.3)	37 (61.7)		
College and above	5 (8.3)	1 (1.7)		
Anesthesia time, (min)	98 (84, 118)	98 (84, 117)	-0.478	0.630

**Notes**: Data for age, BMI, and fasting time are presented as the mean ± standard deviation. Other data are presented as medians (Q1, Q3) or numbers (percentages). No statistically significant differences are observed between the groups

**Abbreviations**: BMI, body mass index; SBP, systolic blood pressure; DBP, diastolic blood pressure; MAP, mean arterial pressure; ASA, American Society of Anesthesiologists

Table 2 shows the incidence of hypotension and the minimum MAP during the induction and maintenance of anesthesia and in the PACU. The incidence of PIH was lower and minimum MAP during induction were higher in the remimazolam group than those in the propofol group. However, there was no significant difference in the incidence of hypotension and the lowest MAP between the two groups during the maintenance of anesthesia and in the PACU. According to the World Health Organization, individuals aged 60 and above are considered elderly. The incidence of PIH in adults with hypertension was lower in the remimazolam group compared to the propofol group, and this difference was significant. However, the difference in elderly patients with hypertension was not significant between the two groups.

**Table 2:** Incidence of hypotension and minimum MAP (n = 60 in each group)

	Propofol group	Remimazolam group	Chi-square value or t-value	*P*-value
Incidence of hypotension n (%)				
During the induction (total)	35 (58.3)	22 (36.7)	5.647	0.017*
During the induction (elderly)	23 (60.5)	17 (45.9)	1.601	0.206
During the induction (adult)	12 (54.5)	5 (21.7)	5.148	0.023*
During the maintenance	31 (51.7)	31 (51.7)	0.000	> 0.99
In the PACU	1 (1.7)	2 (3.4)	0.000	0.988
Minimum MAP (mmHg)				
During the induction	66.7 ± 8.8	71.3 ± 7.7	-3.004	0.003*
During the maintenance	69.4 ± 6.4	70.9 ± 6.3	-1.253	0.210
In the PACU	92.5 ± 10.9	90.6 ± 10.1	0.989	0.330

**Notes**: Data on the incidence of hypotension are presented as numbers (percentages). Data for the minimum MAP are presented as mean ± standard deviation. *P < 0.05, compared with the propofol group

**Abbreviations**: MAP, mean arterial pressure; PACU, post-anesthesia care unit

**Fig. 2:**
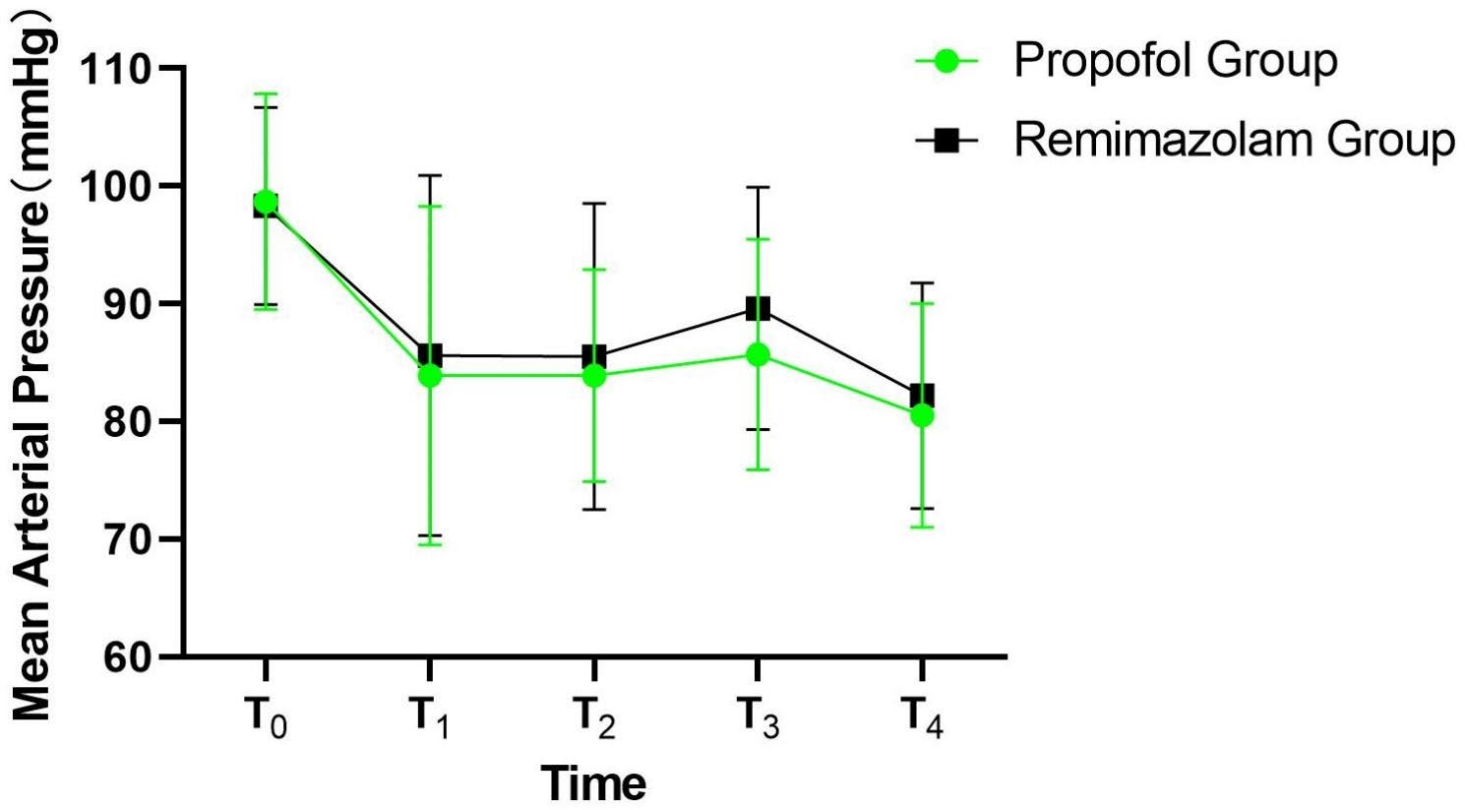
Changes in mean arterial pressure during anesthesia **Notes**: The circles and squares show the mean of mean arterial pressure, and the error bars show the standard deviation of mean arterial pressure **Abbreviations**: T_0_: baseline; T_1_: 10 min after the induction of anesthesia; T_2_: 10 min after the surgery began; T_3_: 30 min after the surgery began; T_4_: 60 min after the surgery began

Table 3; Fig. 2 show the MAP at various times during anesthesia. The overall MAP difference between the two groups could not be considered significant; however, the MAP difference at each time point was significant. Additionally, there was no interaction effect between the group and time; therefore, the MAP cannot be considered the same at different time points. Pairwise comparisons showed that the relation between the MAP at the different times was T_0_ > T_3_ > T_1_=T_2_ >T_4_.

**Table 3:** MAP during anesthesia (n = 60 in each group)

MAP (mmHg)	T_0_	T_1_	T_2_	T_3_	T_4_
Propofol group	98.7 ± 9.2	83.9 ± 14.4	83.9 ± 9.0	85.7 ± 9.8	80.5 ± 9.5
Remimazolam group	98.3 ± 8.4	85.6 ± 15.3	85.5 ± 13.0	89.6 ± 10.3	82.2 ± 9.6
* F*-value _group/time/time × group_		1.986/52.52/0.678			
*P*-value _group/time/time × group_		0.161/<0.001/0.578			

**Notes**: Data for MAP are presented as mean ± standard deviation. T_0_: baseline; T_1_: 10 min after induction; T_2_: 10 min after beginning surgery; T_3_: 30 min after beginning surgery; T_4_: 60 min after beginning surgery

**Abbreviations**: MAP, mean arterial pressure

Table 4 shows the administration of cardiovascular drugs. The frequency of ephedrine application during anesthesia was higher in the propofol group than in the remimazolam group (61.7% in the propofol group vs. 46.7% in the remimazolam group). However, the difference was not significant. There was no significant difference in the frequency of urapidil and atropine use between the two groups.

**Table 4:** Application of cardiovascular active drugs during anesthesia (n = 60 in each group)

	Propofol group	Remimazolam group	Chi-square value	*P*-value
Application of ephedrine n (%)				
During the induction	25 (41.7)	21 (35.0)	0.564	0.453
During the maintenance	25 (41.7)	21 (35.0)	0.564	0.453
Total	37 (61.7)	28 (46.7)	2.719	0.099
Application of urapidil n (%)	5 (8.3)	3 (5.0)	0.134	0.714
Application of atropine n (%)	7 (11.7)	1 (1.7)	3.348	0.067

**Notes**: Data are presented as numbers (percentages)

Adverse events in the PACU and the QoR-40 scores on postoperative day 1 are presented in Table 5. The incidence of nausea and vomiting, blood pressure fluctuation, and QoR-40 scores were not significantly different between the two groups. No cases of emergence delirium or intraoperative awareness occurred during anesthesia recovery in either group.

**Table 5:** Adverse events in the anesthesia recovery period, QoR-40 scores (n = 60 in each group)

	Propofol group	Remimazolam group	Chi-square value	*P*-value
Nausea and vomiting n (%)	1 (1.7)	2 (3.3)	0.000	> 0.99
Fluctuation of blood pressure n (%)	7 (11.7)	8 (13.3)		0.783
Postoperative pain scores	0.5 (0.0, 1.0)	0.5 (0.0, 1.0)	-0.365	0.715
Intraoperative awareness n (%)	0 (0.0)	0 (0.0)	——	——
Emergence delirium n (%)	0 (0.0)	0 (0.0)	——	——
QoR-40 scores	186 (179, 191)	187 (179, 189)	-0.11	0.805

**Notes**: Data for postoperative pain scores and QoR-40 scores are presented as medians (Q1, Q3). Other data are presented as numbers (percentages)

**Abbreviations**: QoR-40, Quality of Recovery-40

## Discussion

In this study, we compared general anesthesia with remimazolam and propofol in terms of PIH in patients undergoing breast cancer surgery. Our results revealed that the remimazolam group had a lower incidence of PIH than that of the propofol group. We used several definitions of hypotension, and although the incidence of hypotension varied between the two groups, the results remained consistent (Supplemental Table 1). It can be concluded that induction with remimazolam in hypertensive patients may result in more stable blood pressure than that with propofol. Studies have reported an association between intraoperative hypotension and mortality far beyond the peri-operative period, with significant associations for 30-day and 1-year mortalities [4, 16, 22, 23]. As the most important part of intraoperative hypotension, PIH is considered an independent risk factor for predicting adverse clinical outcomes. Transient hypotension is associated with tissue hypoperfusion and subsequent complications, such as prolonged intensive care unit stay and postoperative ventilation need, which can increase postoperative mortality [24–27]. Reich et al. [24] advised against propofol induction in patients with a baseline MAP of < 70 mmHg because increasing the dose of propofol increased the risk of PIH. Although many studies have reported that remimazolam is preferred to propofol in hemodynamic stability, they mainly target healthy people and patients undergoing gastroendoscopy and cardiac surgery [28–32], and studies on patients with hypertension have not been reported. Our study observed a significant decrease in the incidence of PIH in adults with hypertension in the remimazolam group compared to the propofol group. However, no significant difference was found between the two groups in elderly patients with hypertension. This lack of significance may be attributed to the insufficient sample size. To obtain more accurate results, it is recommended to increase the sample size for further observations. Finally, we measured the incidence of hypertension during intubation in both groups (Supplementary Table 2) and found no significant difference. Our study demonstrated the hemodynamic benefits of remimazolam in hypertensive populations.

There was no significant difference in hemodynamics between the two groups after the start of surgery, which could be attributed to the adjustment of sevoflurane dosage based on BIS and blood pressure. We believe that remimazolam is safe, efficacious, and non-inferior to propofol for maintaining anesthesia in patients with hypertension.

Our study found no significant difference in the QoR-40 scores between the two groups 1 day after surgery. However, Mao et al. [33] found that patients undergoing urological surgery had lower QoR-15 scores 1 day after surgery in the remimazolam group than in the propofol group. The difference in the quality of recovery between ours and Mao’s studies could be attributed to the different scales evaluated and the population chosen. However, breast cancer surgery is less traumatic, and patients recover well; therefore, the influence of anesthetics on the prognosis is difficult to reflect.

Adverse events recorded in the PACU, including nausea and vomiting, blood pressure fluctuations, intraoperative awareness, and postoperative delirium, were similar between the two groups. It should be noted that these adverse events were secondary outcomes, and the sample size was insufficient. Meanwhile, if the sedation level of remimazolam is more accurately controlled by administering the antagonist flumazenil, the rate of adverse events might change. Therefore, further investigation of the adverse events associated with remimazolam is warranted.

There were some limitations to this study. First, the hypertensive patients chosen were only those undergoing breast cancer surgery. As the surgical trauma of breast cancer surgery is minimal and the patients recover quickly, the influence of anesthetics on patients during and after surgery can be easily concealed. Second, there was no stratification of hypertension in this study, as the sensitivity to anesthetic drugs, incidence of hypotension, and prognosis of patients with different degrees of hypertension may be different. Finally, the follow-up of patients was limited to 1 day, and there was a lack of observation and documentation of long-term complications.

## Conclusion

In conclusion, remimazolam is safe and reliable for general anesthesia in patients with hypertension undergoing breast cancer surgery. The stability of blood pressure during anesthesia induction with remimazolam was better than that with propofol. Remimazolam is a promising agent for general anesthesia in patients with hypertension.

### Electronic supplementary material

Below is the link to the electronic supplementary material.

Supplementary Material 1

## Data Availability

All data generated or analyzed during this study are included in this published article and its supplementary information files.

## Abbreviations

PIH: Post-induction hypotension
MAP: Mean arterial pressure
QoR-40: Quality of Recovery-40 scale
ASA: American Society of Anesthesiologists
BIS: Bispectral index
PACU: Post-anesthesia care unit
