# Supplementary material for: Efficacy and safety of remimazolam compared with propofol in hypertensive patients undergoing breast cancer surgery: a single-center, randomized, controlled study
Source: BMC Anesthesiol. 2023 Dec 12;23:409. doi: 10.1186/s12871-023-02364-x (PMC10714447; doi:10.1186/s12871-023-02364-x)
Supplement: Supplementary file 1 — Supplementary Material 1 [file 12871_2023_2364_MOESM1_ESM.docx]

# Supplementary Materials

**Supplemental Table 1** Incidence of PIH under different definitions (n = 60 in each group)

| Incidence of PIH n (%) | Propofol group | Remimazolam group | Chi-square value | *P*-value |
| --- | --- | --- | --- | --- |
| MAP decreased >30% from baseline | 34 (56.7) | 22 (36.7) | 4.821 | 0.028 |
| MAP decreased >20% from baseline | 53 (88.3) | 42 (70.0) | 6.114 | 0.013 |
| MAP<65 mmHg | 28 (46.7) | 12 (20.0) | 9.600 | 0.002 |
| MAP<60 mmHg | 9 (15.0) | 3 (5.0) | 3.333 | 0.068 |
| MAP decreased >30% from baseline or MAP<65 mmHg | 39 (65.0) | 22 (36.7) | 9.636 | 0.002 |
| MAP decreased >30% from baseline or MAP<60 mmHg | 35 (58.3) | 22 (36.7) | 5.647 | 0.017 |

**Notes:** Data were shown with the number (percentage).

**Abbreviations:** MAP, mean arterial pressure

**Supplemental Table 2** Incidence of hypertension during induction of anesthesia (n = 60 in each group)

|  | Propofol group | Remimazolam group | Chi-square value | *P*-value |
| --- | --- | --- | --- | --- |
| MAP increased >20% from baseline | 13 (21.7) | 9 (15.0) | 0.891 | 0.345 |
| MAP increased >30% from baseline | 2（3.3） | 2（3.3） | 0.000 | >0.99 |

**Notes:** Data were shown with the number (percentage).

**Abbreviations:** MAP, mean arterial pressure
